# Supplementary material for: Bringing the ends together: cryo-EM structures of mycobacterial Ku in complex with DNA define its role in NHEJ synapsis
Source: Nucleic Acids Res. 2026 Jan 9;54(1):gkaf1418. doi: 10.1093/nar/gkaf1418 (PMC12784962; doi:10.1093/nar/gkaf1418)
Supplement: gkaf1418_Supplemental_Files [file gkaf1418_supplemental_files.zip › Supplementary Figures and Tables.pdf]

Supplementary Figures:

Supplementary Figure 1 | Sequencing confirmation of mKu, mKu Ala, mKu 12-15

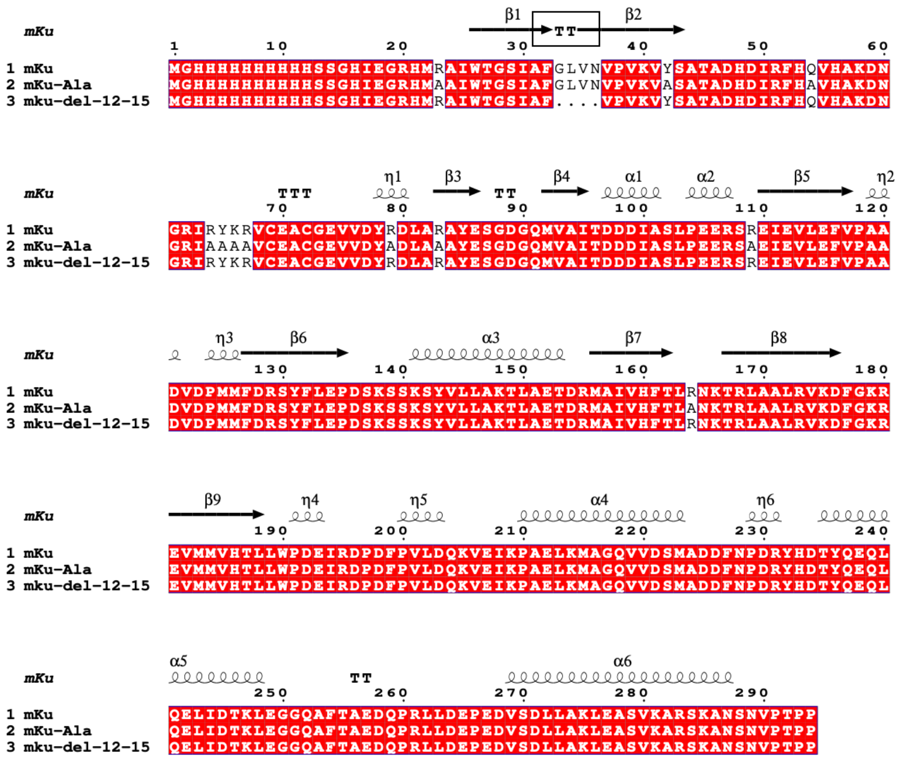

Supplementary Figure 1 | Sequencing confirmation of mKu and its mutants: A topology diagram is shown at the top, aligned with the sequence alignment below. Residues 12–15, which form the loop region of β-hairpin-1, are highlighted with a black box. Eleven residues within the DNA-binding core were substituted with alanine in the DNA-binding–impaired mutant (mKu-Ala).

Supplementary Figure 2 | Purification of mKu and its mutants

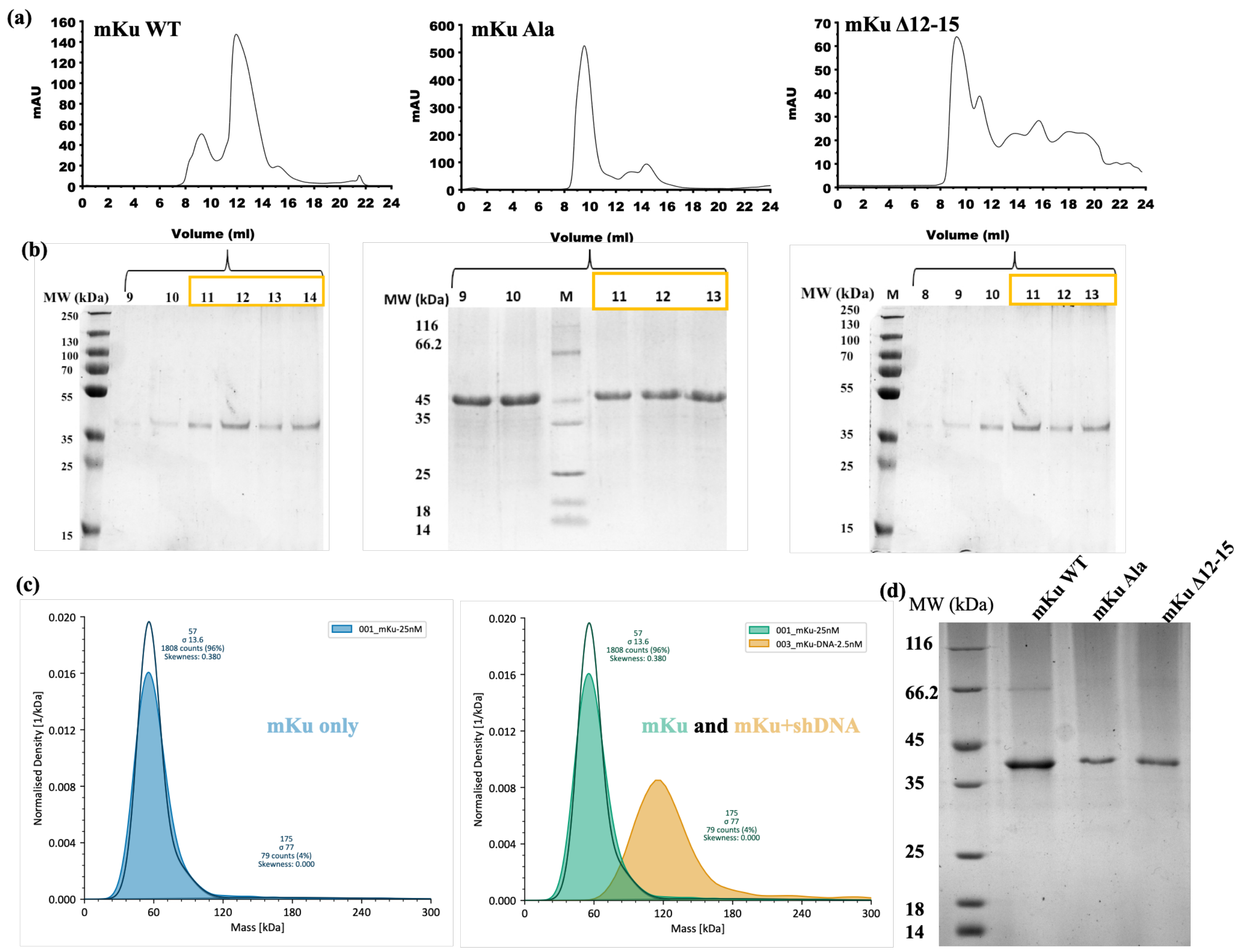

Supplementary Figure 2 | Purification of mKu and its mutants: (a) Analytical size-exclusion chromatography (SEC) profiles of mKu WT, mKu Ala, and mKu Δ12–15 proteins on a Superdex-200 10/300 GL column (Cytiva). Each protein elutes in two phases: an early peak at 8–10 ml with $OD_{260/280} > 1$, and a later peak at 11–14 ml with $OD_{260/280} \leq 0.6$. Both the fractions (early and late) show a single band in SDS PAGE. The early-eluting fractions correspond to higher-order oligomers of mKu bound to endogenous *E. coli* DNA, reflecting the high DNA-binding affinity of mKu. For experimental assays, fractions from 11–14 ml were pooled due to minimal DNA contamination. (b) SDS-PAGE analysis of SEC fractions (9–14 ml) for mKu WT and mutants, confirming protein purity in the pooled fractions. (c) Mass photometry analysis of mKu alone and mKu incubated with short DNA. The protein-only sample shows a molecular weight of ~60 kDa (blue), consistent with the theoretical molecular weight of 66.8 kDa. Upon addition of DNA, the population shifts to ~175 kDa (yellow), indicating formation of mKu–DNA oligomers. The broad peak distribution (mKu+DNA) reflects the heterogeneity of the sample due to higher-order oligomerisation. (d) SDS-PAGE analysis of purified mKu WT and mutant proteins after sequential purification, showing the final samples used for experiments. A faint band at ~66 kDa corresponds to mKu dimers, a characteristic feature of mKu proteins arising from their partial resistance to SDS and high thermal stability(8).

Supplementary Figure 3 | Comparison between mKu-dsDNA and mKu-shDNA complexes

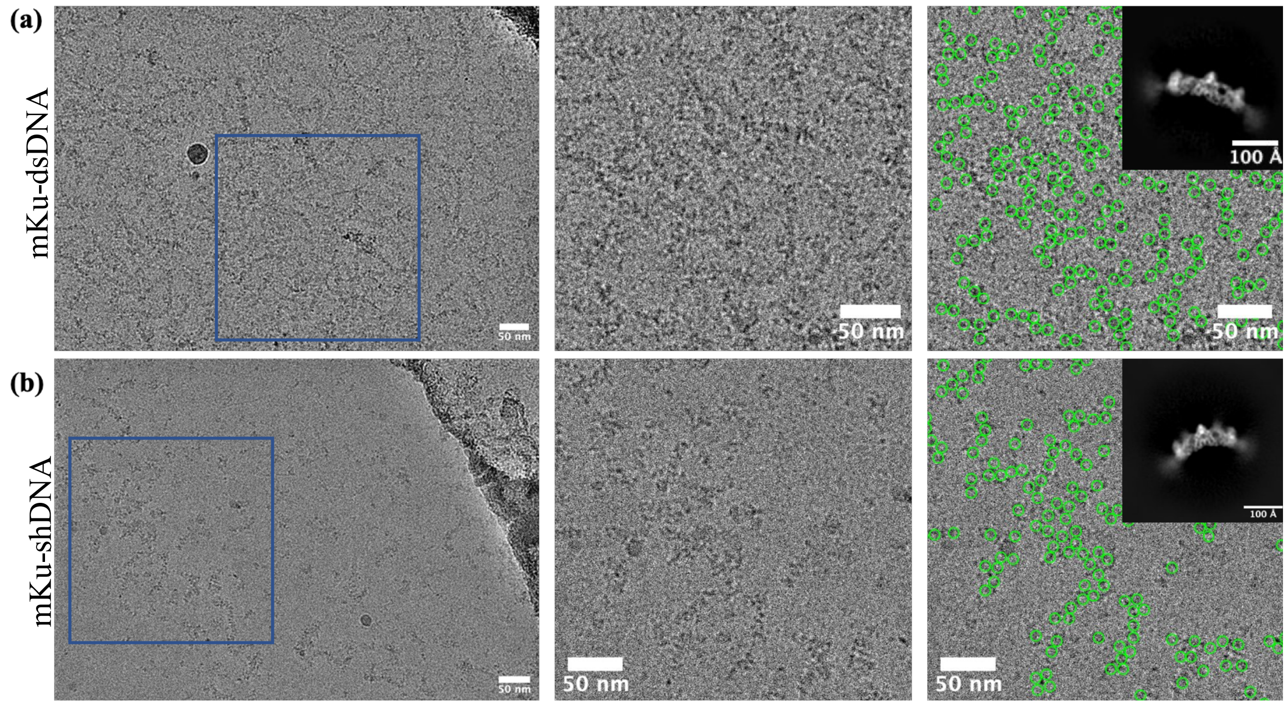

Supplementary Figure 3 | Comparison between cryo-EM datasets of mKu complex: Analysis of the mKu-dsDNA (a) and mKu-shDNA (b) oligomers. The first panel displays the raw cryo-EM micrographs for each complex. The subsequent panels show an enlarged section of the micrographs highlighting the particles, followed by the same section with particle picks marked with green circles (100 Å diameter) to estimate oligomer size. On the rightmost panel, representative 2D class averages illustrate the differences in bending or curvature between the mKu-dsDNA and mKu-shDNA complexes.

Supplementary Figure 4 | Cryo-EM data processing workflow

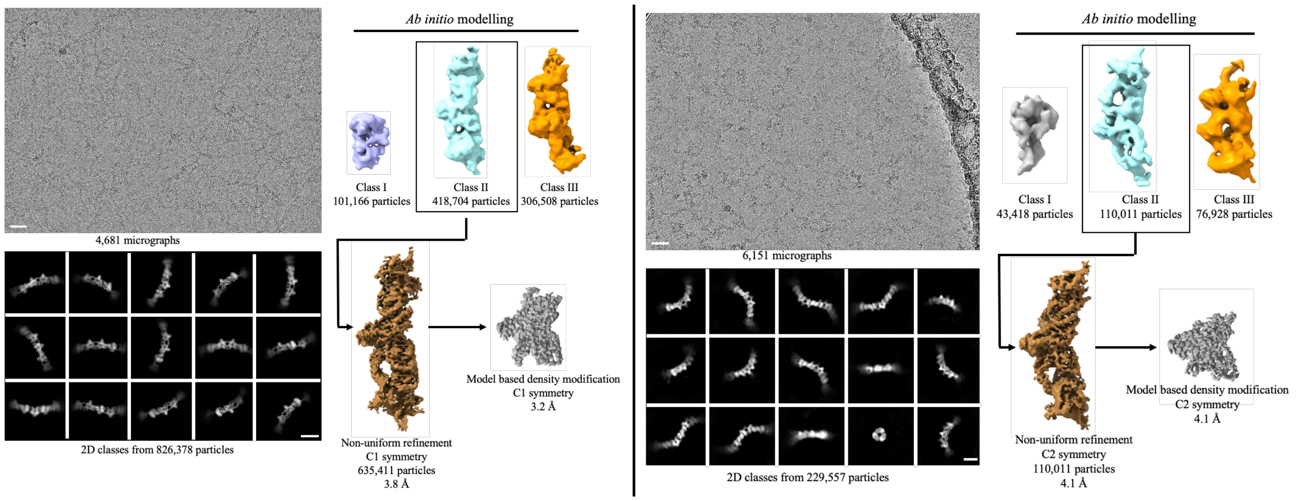

Supplementary Figure 4 | Cryo-EM data processing workflow for mKu-DNA complexes: The left and right panels illustrate the data processing workflows for the mKu-dsDNA and mKu-shDNA datasets, respectively. Each workflow sequentially presents the raw micrographs, 2D class averages, ab initio models, and refined models, showcasing the progression from raw data to high-resolution structures.

Supplementary Figure 5 | Map validation and resolution estimation

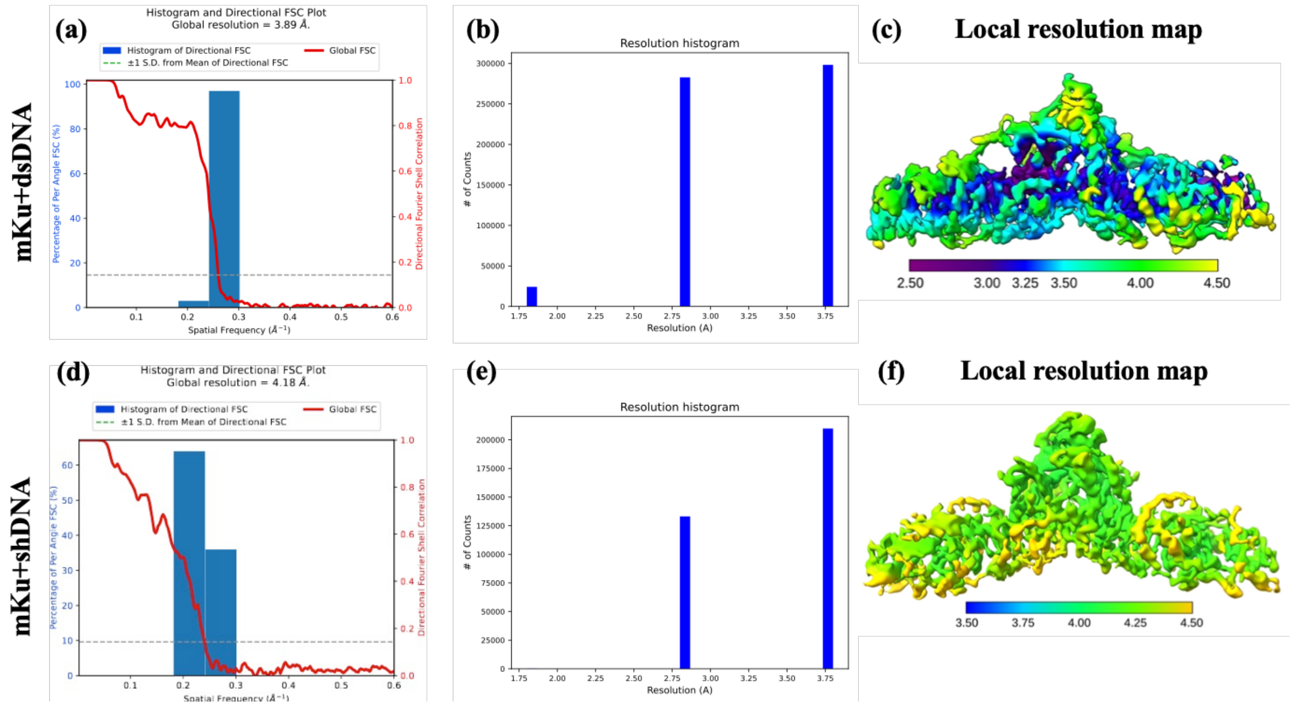

Supplementary Figure 5 | Map validation and resolution estimation for mKu-DNA complexes: (a & d) 3D Fourier Shell Correlation (FSC) plot from CryoSparc showing the global resolution of the map; (b & e) resolution histogram generated by ResMap; (c & f) local resolution map calculated using Phenix, indicating the range of resolution values across the respective maps.

Supplementary Figure 6 | FRET showing mKu-mediated DNA synapsis

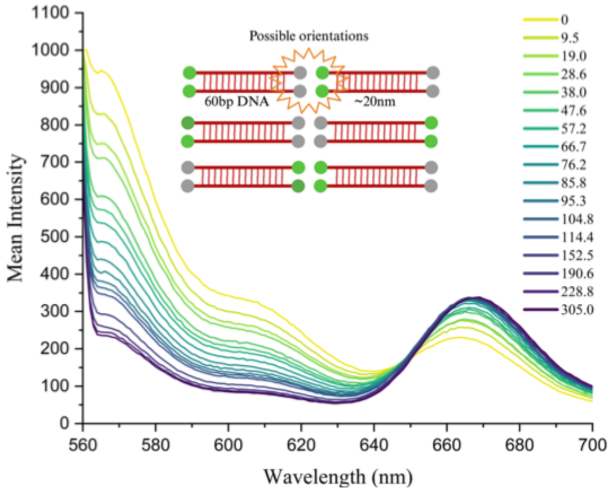

Supplementary Figure 6 | FRET analysis of DNA substrate interactions in the presence of mKu: Wavelength vs. fluorescence intensity plot illustrating Förster Resonance Energy Transfer (FRET) between DNA substrates labelled with donor (Cy3) and acceptor (Cy5) fluorophores. The DNA concentration was fixed at 250 µM, while mKu was titrated incrementally from 0 to 305 µM. The schematic representation depicts the synaptic orientations enabling successful FRET, with the donor fluorophore shown in green and the acceptor in grey.

Supplementary Figure 7 | Live cell imaging captures post-DNA damage response of mKu

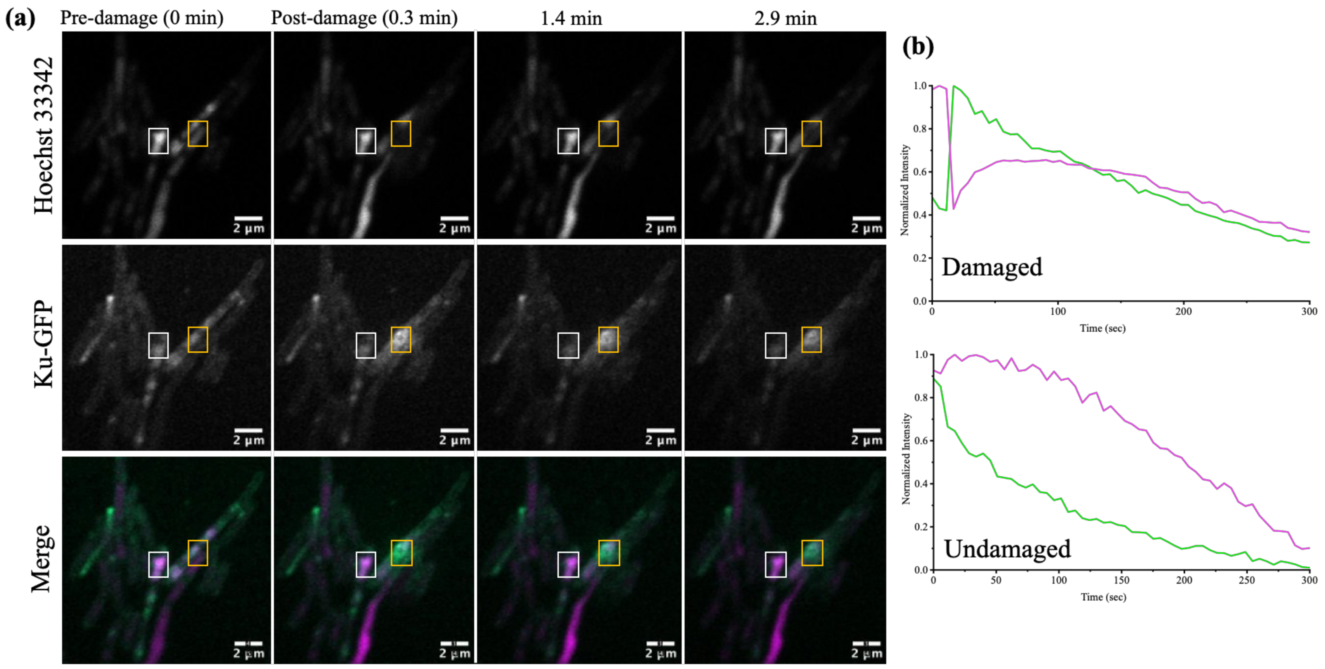

Supplementary Figure 7 | Live cell imaging captures DNA damage response of mKu: (a) Dynamics of mKu-GFP shown pre- and post-laser-induced DNA damage. The laser damage is focused within a 0.8 µm x 1 µm region (yellow box); the control ROI has the same dimensions (white) but no laser damage. The dimensions of the boxes are not scaled. DNA is stained with Hoechst 33342. (b) Intensity quantification over time, with magenta and green lines representing the dynamics of DNA and mKu-GFP signals at damaged and undamaged ROI, respectively.

Supplementary Figure 8 | mKu-mKu dimer interface

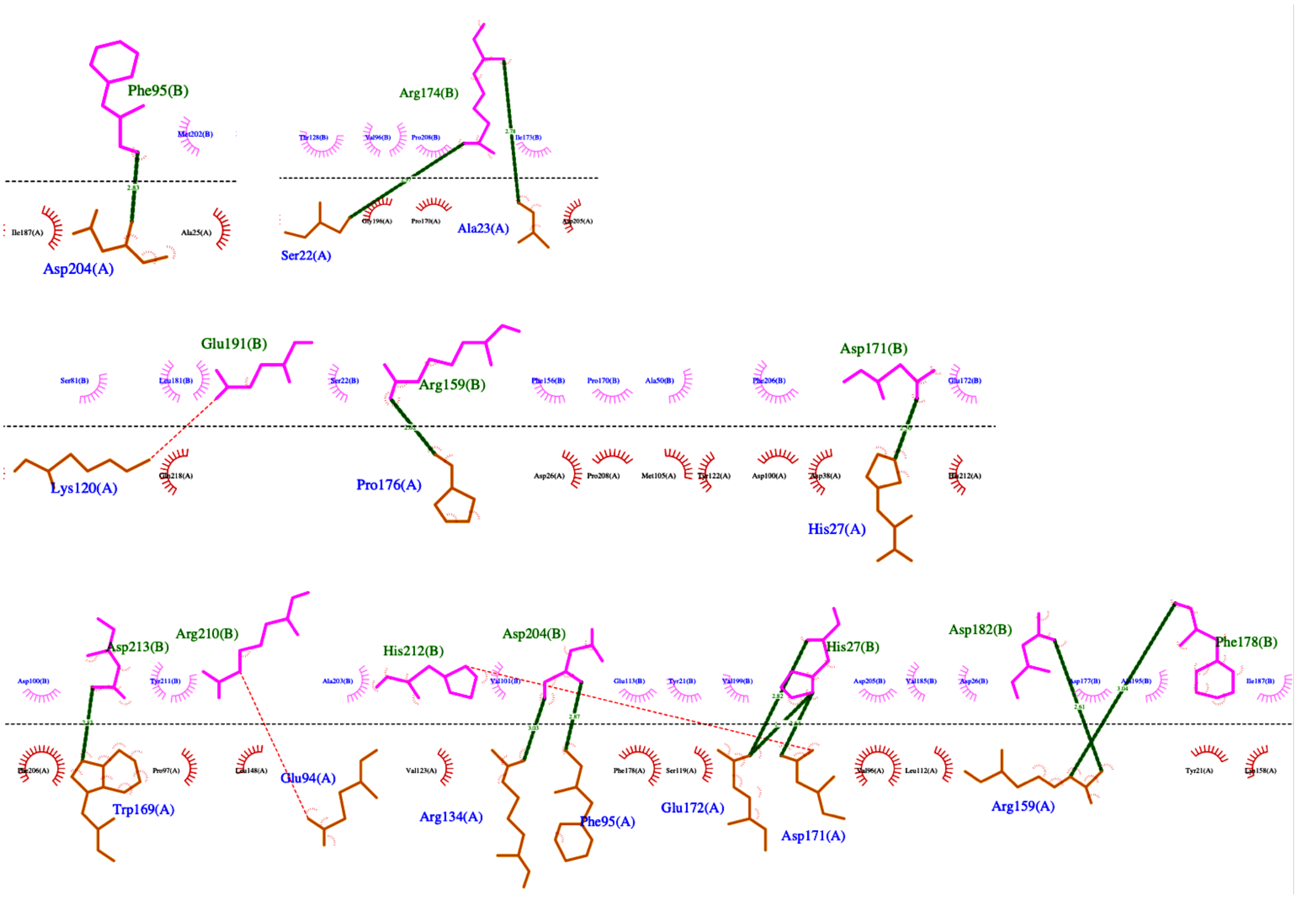

Supplementary Figure 8 | Protein-protein dimerization interface of the mKu dimer: The dimerization interface is depicted using a 2D contact map generated with LigPlot+. Amino acid residues from the two monomeric chains are

coloured purple and brown, respectively. Neighbouring hydrophobic residues are represented as spikes. Hydrogen bonds are shown as green dashed lines, while salt bridges are indicated with red dashed lines.

Supplementary Figure 9 | The Core DNA-binding domain of Ku is structurally conserved

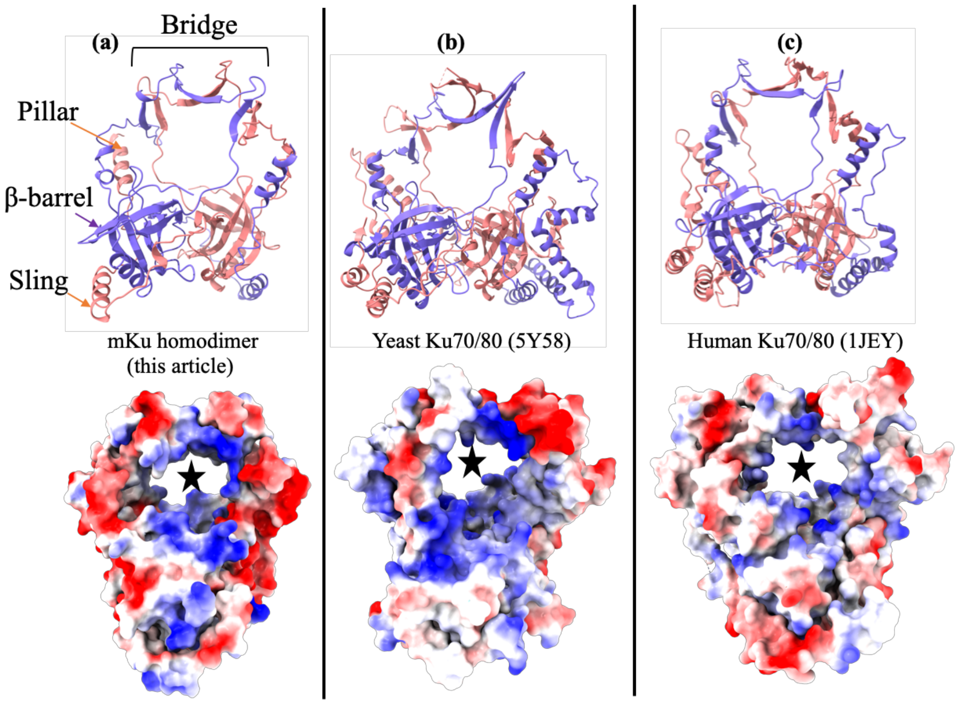

Supplementary Figure 9 | Comparison of the core DNA-binding domain of Ku proteins: Experimentally determined structures reveal the similarity of the DNA-binding domain across mKu (PDB: 8VF5; this study) (a), yeast Ku70/80 (PDB: 5Y58) (b), and human Ku70/80 (PDB: 1JEY) (c). Additional domains in yeast and human Ku were removed for clarity. The bottom panels illustrate the electrostatic charge distributions, with positive to negative charges represented by red to blue gradients. The DNA-binding pores, lined with positively charged residues, are marked with a star symbol.

Supplementary Figure 10 | Comparison of DNA binding residues of Ku (Mtb, Human and Yeast)

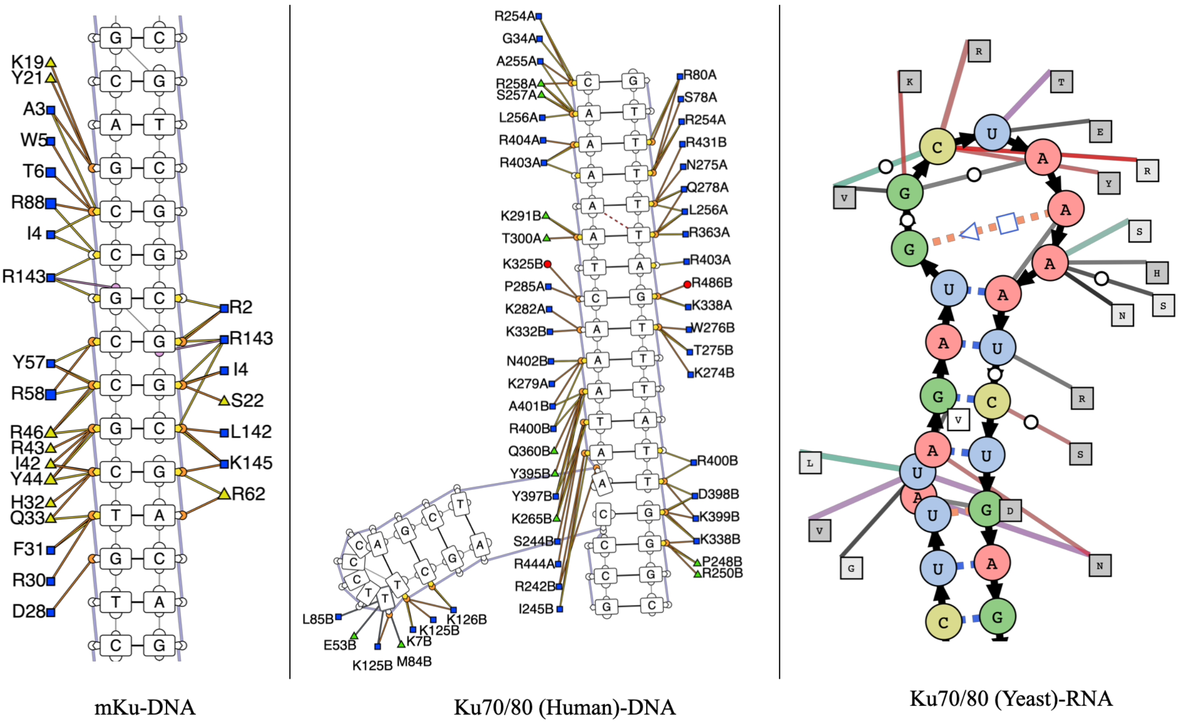

Supplementary Figure 10 | Comparison of the DNA-binding residues of Ku proteins: Experimentally determined structures highlight the DNA-binding residues of mKu (PDB: 8VF5; this study), human Ku70/80 (PDB: 1JEY) and yeast

Ku70/80 (PDB: 5Y58). The yeast Ku70/80 structure is shown in complex with RNA. Despite the absence of sequence homology at the DNA/RNA-binding pore, the binding mode is conserved, with most contacts mediated by positively charged residues (Arg and Lys) interacting with the sugar–phosphate backbone.

Supplementary Figure 11 | Predictive structural modelling of mKu Ala (12-15) DNA synaptic complex

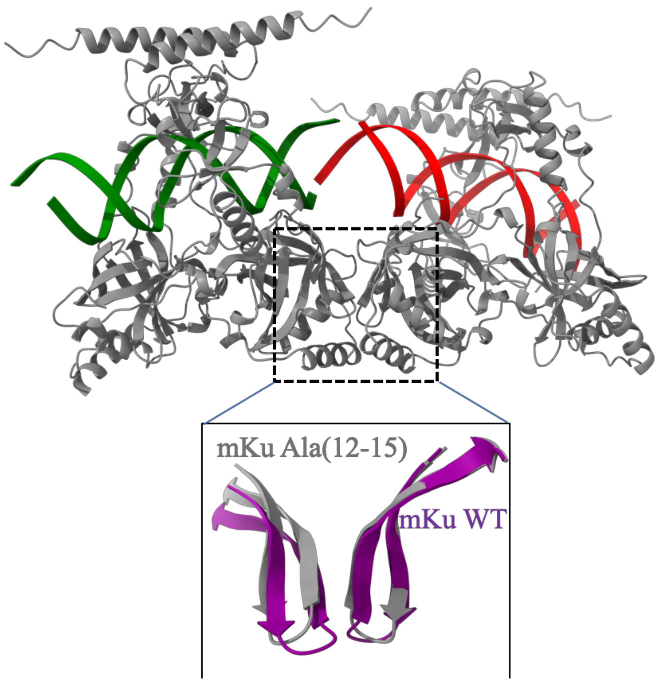

Supplementary Figure 11 | Predictive structural modelling of the mKu Ala (12–15) DNA synaptic complex: AlphaFold3-predicted model of the mKu Ala (12–15) mutant bound to two dsDNA duplexes. The synaptic interface remains intact despite alanine substitution, in contrast to the Δ (12–15) mutant, where synapsis is disrupted. This suggests that the overall β-hairpin 1 topology, rather than specific side-chain interactions, is the primary determinant of synapsis. The β-hairpin 1 of WT (purple) and Ala (12–15) (grey) mKu are superimposed to illustrate deviations from the wild-type synaptic conformation.

Supplementary Tables:

Supplementary Table 1 | Composition of buffers used in this study

Buffer	Composition
Resuspension buffer	50 mM Tris pH 7.5, 250 mM NaCl, 10% sucrose, PMSF 17 $\mu g \cdot ml^{-1}$, benzamidine 34 $\mu g \cdot ml^{-1}$
IMAC wash buffer-1	50 mM Tris pH 7.5, 400 mM NaCl, 10 mM imidazole, 10% glycerol, PMSF 17 $\mu g \cdot ml^{-1}$, benzamidine 34 $\mu g \cdot ml^{-1}$
IMAC wash buffer-2	50 mM Tris pH 7.5, 400 mM NaCl, 45 mM imidazole, 10% glycerol, PMSF 17 $\mu g \cdot ml^{-1}$, benzamidine 34 $\mu g \cdot ml^{-1}$
IMAC elution buffer	50 mM Tris pH 7.5, 400 mM NaCl, 300 mM imidazole, 10% glycerol, PMSF 17 $\mu g \cdot ml^{-1}$, benzamidine 34 $\mu g \cdot ml^{-1}$
IEX buffer-1	50 mM Tris pH 7.5, 50 mM NaCl, 2 mM DTT, 10% glycerol, PMSF 17 $\mu g \cdot ml^{-1}$, benzamidine 34 $\mu g \cdot ml^{-1}$
IEX buffer-2	50 mM Tris pH 7.5, 1 M NaCl, 2 mM DTT, 10% glycerol, PMSF 17 $\mu g \cdot ml^{-1}$, benzamidine 34 $\mu g \cdot ml^{-1}$
SEC buffer	25 mM HEPES pH 7.5, 150 mM NaCl, 2 mM DTT, 10% glycerol, PMSF 17 $\mu g \cdot ml^{-1}$, benzamidine 34 $\mu g \cdot ml^{-1}$

Supplementary Table 2 | Cryo-EM data collection, refinement, and validation statistics

	mKu-linear DNA		mKu-hairpin DNA	
	Unit complex	Supercomplex	Unit complex	Supercomplex
**Data collection and processing**	PDB: 8VF5<br>EMD: 43186	PDB: 8VF2<br>EMD: 43184	PDB: 8V53<br>EMD: 42978	PDB: 8VF4<br>EMD: 43185
Micrographs	6151		4682	
Magnification	105,000		105,000	
Voltage (kV)	300		300	
Electron exposure ($e^{-}/Å^2$)	40		40	
Defocus range (μm)	-1.5 to -2.5		-1.5 to -2.5	
Pixel size (Å)	0.833		0.833	
Symmetry imposed	C1		C2	
Initial particle (no.)	~ 4,800,000		~ 1,150,000	
Final particle (no.)	635,411		110,011	
Map resolution (Å)	3.7	3.7	4.1	4.1
FSC threshold	0.143	0.143	0.143	0.143
Map resolution range (Å)	2.0-3.9	2.0 - 5.7	4.0 - 5.2	4.0 - 5.2
**Refinement**				
Model resolution (Å)	3.7	3.7	4.1	4.1
FSC threshold	0.143	0.143	0.143	0.143
Map sharpening B factor ($Å^2$)	168.6	168.6	161.5	161.5
**Model composition**				
Non-hydrogen atoms	4513	13,367	4281	12,777
Protein residues	453	1359	453	1359
DNA	42	118	31	90
**B factors ($Å^2$)**				
Protein	65.04	84.86	150.25	143.92
DNA	75.53	153.25	139.88	216.43
**R.M.S.D.**				
Bond lengths (Å)	0.004	0.004	0.004	0.004
Bond angles (°)	0.91	0.89	0.92	0.89
**Validation**				
MolProbity score	1.95	1.9	1.96	1.99
Clash score	10.39	11.48	14.58	16.72
Rotamer outliers (%)	0.25	0.34	0.76	0.42
**Ramachandran plot**				
Favoured (%)	93.76	95.4	95.77	96.14
Allowed (%)	6.24	4.6	4.23	3.86
Disallowed (%)	0	0	0	0

Supplementary Table 3 | List of interacting residues for mKu-mKu dimer

Atom no.	Atom name	Res name	Res no.	Chain ID		Atom no.	Atom name	Res name	Res no.	Chain ID	Distance (Å)
302	N	Ala	61	B	---	169	O	Val	34	A	2.194
355	N	Arg	41	A	---	187	OE2	Glu	49	B	2.334
126	N	His	32	A	---	211	O	Ala	63	B	2.476
326	OH	Tyr	211	A	---	266	OE1	Glu	94	B	2.477
135	NE2	His	32	A	---	385	OE2	Glu	65	B	2.496
443	NE2	His	27	A	---	556	OD2	Asp	171	B	2.496
455	NH2	Arg	210	A	---	267	OE2	Glu	94	B	2.541
713	NZ	Lys	120	B	---	722	OE2	Glu	191	A	2.571
115	NH2	Arg	159	A	---	393	OD2	Asp	182	B	2.61
589	NH1	Arg	159	B	---	477	O	Pro	176	A	2.622
454	NH1	Arg	210	A	---	267	OE2	Glu	94	B	2.627
74	N	Glu	49	A	---	147	O	Arg	41	B	2.653
83	N	Val	47	B	---	200	O	Arg	43	A	2.725
154	NH2	Arg	41	B	---	195	OE1	Glu	65	A	2.755
122	ND1	His	27	B	---	104	OE2	Glu	172	A	2.757
213	N	Val	34	B	---	248	O	Ala	61	A	2.766
234	N	Arg	174	B	---	497	O	Ala	23	A	2.781
197	N	Arg	43	A	---	86	O	Val	47	B	2.791
279	N	Ala	63	A	---	67	O	His	32	B	2.8
116	N	His	27	B	---	104	OE2	Glu	172	A	2.817
466	N	Asp	204	A	---	531	O	Phe	95	B	2.826
23	NE1	Trp	169	A	---	143	OD2	Asp	213	B	2.833
122	ND1	His	27	B	---	353	OD1	Asp	171	A	2.842
208	N	Ala	63	B	---	129	O	His	32	A	2.866
307	N	Asp	204	B	---	602	O	Phe	95	A	2.867
64	N	His	32	B	---	282	O	Ala	63	A	2.867
245	N	Ala	61	A	---	216	O	Val	34	B	2.891
38	NH1	Arg	43	B	---	54	O	Val	47	A	2.894
244	NH2	Arg	174	B	---	1055	OG	Ser	22	A	2.973
51	N	Val	47	A	---	32	O	Arg	43	B	3.029
704	NH2	Arg	134	A	---	310	O	Asp	204	B	3.034
112	NE	Arg	159	A	---	43	O	Phe	178	B	3.037
29	N	Arg	43	B	---	54	O	Val	47	A	3.059
508	NE2	His	35	A	---	421	SG	Cys	51	B	3.062
9	NE1	Trp	169	B	---	233	OD2	Asp	213	A	3.069
618	NZ	Lys	37	A	---	802	OE2	Glu	53	B	3.105
166	N	Val	34	A	---	305	O	Ala	61	B	3.213
275	NE	Arg	46	B	---	405	OH	Tyr	44	A	3.306

Supplementary Table 4 | Impact of mutation of the critical residues in the DNA-protein interface

Chain	Wild Residue	Residue Position	Mutant Residue	RSA(%)	Predicted ΔΔG	Outcome
A	Y	21	A	11.2	-0.115	Destabilizing
A	Q	33	A	16.4	-2.616	Highly destabilizing
A	R	43	A	37.7	-2.432	Highly destabilizing
A	Y	44	A	14.3	-2.665	Highly destabilizing
A	R	46	A	12.1	-4.149	Highly destabilizing
A	R	58	A	77.5	-1.826	Destabilizing
A	R	62	A	32.1	-2.702	Highly destabilizing
A	R	66	A	44.9	-0.869	Destabilizing
B	Y	21	A	11.2	-0.322	Destabilizing
B	Q	33	A	16.4	·2.258	Highly Destabilizing
B	R	43	A	37.7	-3.498	Highly Destabilizing
B	Y	44	A	14.3	-3.427	Highly Destabilizing
B	R	46	A	12.1	-3.338	Highly Destabilizing
B	R	58	A	77.5	-1.566	Destabilizing
B	R	62	A	32.1	-3.046	Highly Destabilizing
B	R	88	A	44.9	-0.934	Destabilizing

Supplementary Table 5 | Impact of mutation of the critical residues in the synaptic interface

Chain	Wild Residue	Residue Position	Mutant Residue	RSA(%)	Predicted ΔΔG	Outcome
A	G	12	A	0.3	-0.563	Destabilizing
A	L	13	A	3.9	-1.919	Destabilizing
A	V	14	A	7.7	-1.653	Destabilizing
A	N	15	A	0.2	-1.571	Destabilizing
F	G	12	A	0.3	-0.464	Destabilizing
F	L	13	A	3.9	-2.15	Highly destabilizing
F	V	14	A	7.7	-2.071	Highly destabilizing
F	N	15	A	0.2	-0.122	Destabilizing

Supplementary Table 6 | HDX-MS data statistics

	**mKu**	**mKu + dsDNA**	**mKu + shDNA**
ligand	-	linear dsDNA 40bp	Hairpin DNA (34 & 21 bp)
[mKu] (μM)	15	15	15
[DNA] (μM)	15	15	15
HDX buffer	5mM K2HPO4, 5mM KH2PO4 pH 7		
HDX time	0.1, 1, 10 & 100 min at 25°C		
HDX control samples	mKu		
Significance cut-offs	ΔHDX > 0.5 Da and *P*-value < 0.01in Welch's *t*-test (*n*=3)		
No. of peptides	208	208	208
Sequence coverage (%)	93.22	93.22	93.22
Average peptide length	11.02	11.02	11.02
Peptide redundancy	8.34	8.34	8.34
Technical replicates	3	3	3
Mean SD	0.087	0.089	0.090
Data availability	PRIDE: PXD060776		
